# *Announcement*: Community Preventive Services Task Force Finding for Year-Round Schooling to Increase Health Equity

**DOI:** 10.15585/mmwr.mm6640a7

**Published:** 2017-10-13

The Community Preventive Services Task Force (CPSTF) recently posted new information about its finding of insufficient evidence to determine the effectiveness of year-round schooling in improving academic achievement. The information for “Health Equity: Year-Round Schooling” is available at https://www.thecommunityguide.org/findings/health-equity-year-round-schooling.

Established in 1996 by the U.S. Department of Health and Human Services, the CPSTF is an independent, nonfederal panel of public health and prevention experts whose members are appointed by the director of CDC. The CPSTF provides information for a wide range of persons who make decisions about programs, services, and other interventions to improve population health. Although CDC provides administrative, scientific, and technical support for the CPSTF, the recommendations developed are those of the task force and do not undergo review or approval by CDC.

